# Dynamic Acoustic Holography: One-Shot High-Precision and High-Information Methodology

**DOI:** 10.3390/mi15111316

**Published:** 2024-10-29

**Authors:** Zhaoxi Li, Yiheng Yang, Qi Lu, Xiongwei Wei, Chenxue Hou, Yi Quan, Xiaozhou Lü, Weimin Bao, Yintang Yang, Chunlong Fei

**Affiliations:** 1 School of Microelectronics, Xidian University, Xi’an 710071, China; 22111213919@stu.xidian.edu.cn (Y.Y.); 20009102129@stu.xidian.edu.cn (Q.L.); 24111110105@stu.xidian.edu.cn (X.W.); houchenxue@xidian.edu.cn (C.H.); quanyi@xidian.edu.cn (Y.Q.); ytyang@xidian.edu.cn (Y.Y.); 2 School of Aerospace Science and Technology, Xidian University, Xi’an 710071, China; xzlu@xidian.edu.cn (X.L.); baoweimin@cashq.ac.cn (W.B.)

**Keywords:** dynamic acoustic holography, acoustic field control, ultrasound array

## Abstract

Acoustic holography technology is widely used in the field of ultrasound due to its capability to achieve complex acoustic fields. The traditional acoustic holography method based on single-phase holograms is limited due to its inability to complete acoustic field control with high dynamics and accuracy. Here, we propose a method for constructing an acoustic holographic model, introducing an ultrasonic array to provide dynamic amplitude control degrees of freedom, and combining the dynamically controllable ultrasonic array and high-precision acoustic hologram to achieve the highest acoustic field accuracy and dynamic range. This simulation method has been proven to be applicable to both simple linear patterns and complex surface patterns. Moreover, it is possible to reconstruct the degree of freedom of the target plane amplitude effectively and achieve a breakthrough in high information content. This high-efficiency acoustic field control capability has potential applications in ultrasound imaging, acoustic tweezers, and neuromodulation.

## 1. Introduction

Acoustic holographic methods demand the precise manipulation of complex acoustic fields within three-dimensional spaces and are used in many fields such as 3D displays [1], acoustic tweezers [2,3,4,5], and ultrasound neuromodulation [6]. These techniques store the spatial information of a wavefront’s phase and amplitude, enabling its reconstruction through interference when exposed to a coherent source [7,8]. Traditional phase acoustic holograms are currently widely used because they are cheap, fast to prepare, and can be used in large-scale and high-power working conditions [9]. However, the phase-only acoustic hologram has the following two shortcomings due to limitations in its degree of freedom and process accuracy. First, its reconstruction efficiency on the target plane is low. The acoustic hologram is obtained by 3D printing. In practical applications, when the printing accuracy is insufficient, it will not match the set target. The patterns differ greatly, especially when it is difficult to accurately reproduce the target pattern amplitude reconstruction distribution. Secondly, the dynamic control effect is poor [10,11]. Once the acoustic hologram is printed and prepared, it is difficult to ensure the high-quality realization of multiple target patterns.

At present, many researchers have conducted a lot of research on the optimization methods of acoustic holography. High reconstruction efficiency in acoustic holography requires the simultaneous control of phase and amplitude modulation [12]. One researcher increased the degree of freedom by superimposing two acoustic holograms to achieve high-quality 2D acoustic field distribution [13]. Computer-generated holograms can efficiently, and with high quality, achieve the distribution of complex acoustic fields through optimization algorithms, such as the DS (Direct Search) method and IASA (Iterative Angular Spectrum Approach), thereby enhancing the effectiveness of sound field control and particle manipulation [14,15]. Compared with traditional sound holograms, the number of reconstructed images (multiple planes) is increased by introducing artificial intelligence and neural networks [16]. Despite this, it is still difficult to achieve high reconstruction and highly dynamic complex acoustic fields at the same time by simply relying on the thickness of the hologram element to change the phase of sound wave transmission. When the sound wave frequency increases or the number of acoustic field planes increases, this restricts the effectiveness of the phase-only acoustic hologram in real-time acoustic field control. The ultrasonic array can control the amplitude and phase of each array element as well as, to a large extent, the sound waves [17,18,19,20]. Other studies have used 8 × 8 ultrasonic array elements to achieve dynamic acoustic field control. However, the difficulty of preparing high-frequency multi-element arrays and the complexity of multi-channel signal excitation (dual control of amplitude and phase) systems have become limitations in the construction of complex and high-precision acoustic field modes. It is far more difficult to increase the number of effective array elements and the number of lens elements. Moreover, Melde et al. used linear array and acoustic holography to dynamically move the acoustic focus and achieved changes in the directivity of the acoustic beam by introducing phase changes in the linear array [9]. However, there are few reports on ultrasound array harmonic holography for complex acoustic fields [21], and this method has not yet been used in 3D acoustic fields and time-varying acoustic fields.

**Figure 1:**
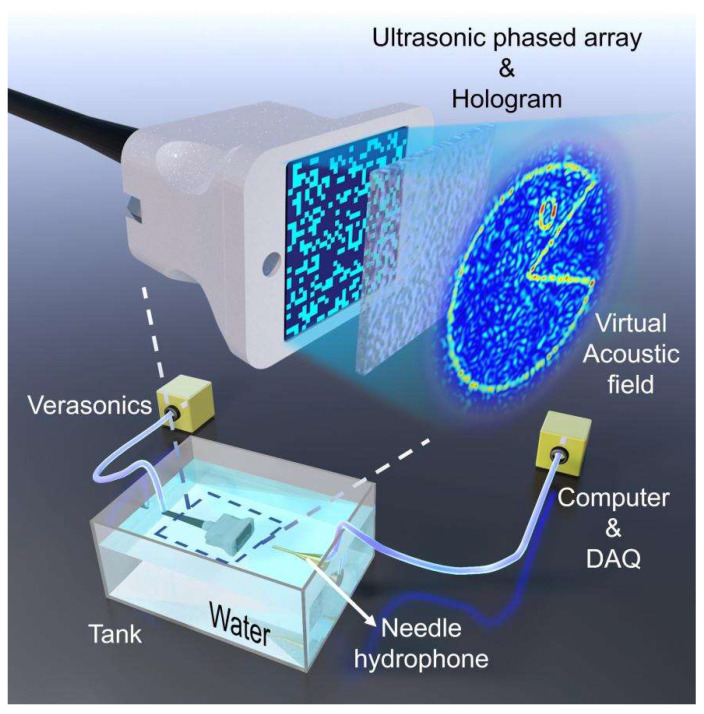
Diagram of the control system of the electric machine and the equipment to be tested.

This paper proposes a new acoustic holographic design method based on dynamic array acoustic field control, which combines the advantages of array dynamic control with the high-resolution advantages of acoustic holograms. Based on the amplitude optimization superposition array acoustic holography algorithm, the reconstruction efficiency of the conventional acoustic holography is improved. Then, the 3D reconstruction of highly active information is realized by using the random selection array acoustic holography algorithm. The schematic diagram of dynamic sound field control using ultrasonic array and acoustic holography is shown in Figure 1. Simulation verification was conducted using two types of arrays (128-element 5.5 MHz line array and 8 MHz 32 × 32 2D array). This method broadens the application field of acoustic holography, aims at more efficient and dynamic complex acoustic field control, and lays the foundation for the application of ultrasound in biomedicine and industry.

## 2. Materials and Methods

### 2.1. Materials

Arrays: In this study, two types of arrays are used for simulation verification. One is a 128-element 5.5 MHz line array, and the other is an 8 MHz 32 × 32 2D array.

Software and Equipment: In future work, the Verasonics system is expected to be used to generate a pulse excitation signal, which is then applied to ultrasonic transducers to generate ultrasonic pressure fields and, the motion scanning acquisition system is based on LabVIEW 2015 software combined with a stylus hydrophone.

Materials of hologram: The hologram for this work were simulated with the GR rigid resin by the 3D printer NanoArch S140(BMF Precision Tech Inc., Chongqing, China). Its speed of sound is measured to be 2400 m/s, with a density of 1300 kg/cm^2^.

### 2.2. Amplitude Optimization for Array Acoustic Holography

In the common use of acoustic holography combined with transducers, all array elements are turned on by default, and the amplitudes of the array elements are made consistent, such that the effect is similar to that of a single-element transducer. The initial thickness of the acoustic hologram is set to $T_{0}$, while $∆T\left(x,y\right)$ is the relative thickness difference,$\;\Delta T\left(x,y\right)=\frac{\Delta \phi (x,y)}{k_{m}-k_{h}}$, $∆\emptyset \left(x,y\right)$ is the phase change compared to the initial plane, $k_{h}$ and $k_{m}$ are the wave numbers in the hologram body and its surrounding medium, respectively, and the subscripts *h* and *m* represent the materials of the hologram and the medium, respectively. Then, the phase on the source surface is calculated through the general iterative angular spectrum approach (IASA) [9] and, finally, the thickness and phase conversion formula is used.

(1) $$T\left(x,y\right)=T_{0}-\Delta T$$

We first obtained the final acoustic holographic thickness distribution and converted it into the model required for 3D printing [22,23,24]. To achieve higher-precision target acoustic field control in this work, we took advantage of the adjustable excitation of each element of the array and adjusted the initial source plane excitation. We used the reverse propagation formula from image to hologram $P(k_{x},k_{y},0)=P(k_{x},k_{y},z)H(k_{x},k_{y},-z)$ to transfer the designed acoustic field on the target surface back to the source plane, where $P(k_{x},k_{y},z)$ represents the angular spectrum of the sound wave at depth *z* in the plane, $P(k_{x},k_{y},0)$ represents the angular spectrum information of the sound hologram plane, $H(k_{x},k_{y},z)$ is the propagation function, and $H(k_{x},k_{y},-z)$ represents the reverse propagation from the target plane to the sound hologram plane, $H(k_{x},k_{y},z)=e^{jz\sqrt{k^{2}-k_{x}^{2}-k_{y}^{2}}}$.

Furthermore, the phase information transmitted to the source plane is retained, while the amplitude information undergoes pixelization corresponding to the amplitude. Specifically, based on the number of elements in the source plane line array or phased array, and the kerf and pitch, the amplitude is divided into several regions. The average amplitude of each region is then taken, followed by a unified normalization process for each region, thereby obtaining new excitation values for each element.

Subsequently, with the initial phase and adjustments, the phase information of the final acoustic hologram is computed using the IASA. It is worth noting that during the inversion process, we do not divide the phase similar to the kerf division of the array, aiming to reduce the 3D printing difficulty caused by element division. Subsequently, the phase information is converted into the corresponding acoustic hologram model.

### 2.3. Achieving Multiple Acoustic Fields Through Monolithic Sound Holography with Random Array Amplitude Distribution

For a conventional acoustic hologram, it is impossible to form multiple different precise acoustic fields under the premise of fixed focusing depth, especially when there is an overlap at the focal points of these acoustic fields, which is the current limitation of traditional acoustic holography. In this work, we propose to form multiple target acoustic fields by selectively utilizing partial array elements each time. However, the simple activation of all the odd or even array elements of a linear array, or pure adjacent element excitation of phased array with a pattern of 0101, results in severe artifacts in the combined acoustic field with acoustic holography, making it impossible to achieve precise control of the acoustic field in a true sense.

Therefore, we draw inspiration from the spatial multiplexing principle in the field of optics, storing the source plane phases of multiple target acoustic fields in a single acoustic hologram to achieve information compression. This method has been proven in optics to generate beams with negligible sidelobes. By selectively activating partial array elements each time, the entire focusing target is achieved. Specifically, all *M* array elements are evenly divided into *N* portions, where *N* represents the number of different acoustic fields at target depths. The number of elements opened each time is n=M/N. The positional relationships between the *n* elements are uncorrelated and variable, exhibiting complete randomness. However, the positions allocated for the *N* groups of elements must not overlap and should correspond one-to-one. After allocating the corresponding array elements for each acoustic field, only the selected elements are used as excitation sources. IASA calculations are then performed, resulting in phase information provided only at the excitation element. The phase information of the *N* elements is superimposed to form the new acoustic holographic phase information.

As for the allocation of array element excitation amplitudes, there are two methods. One method is to simply set the amplitude at the excited elements to “1”, while setting the unselected elements to “0”. Another method is based on precise amplitude control mentioned earlier, where each array element excitation is different, considering the selection of elements.

### 2.4. Quality Evaluation of Hologram Target Acoustic Field

The effectiveness of the reconstructed image can be better evaluated by correlating the sound power of the target area in the acoustic field with the sound power of the whole image [25,26,27]. Therefore, the overall reconstruction efficiency *η* is used to represent the quality of acoustic field reconstruction. According to the relation of sound pressure, sound intensity and sound power, the formula for calculating sound power can ultimately be expressed in terms of sound pressure, as given by the following equation:

(2) $$P_{Z}=\Delta x\Delta y\sum_{(i,j)\in I}I_{z,(i,j)}=\Delta x\Delta y\sum_{(i,j)\in I}\frac{p_{z,(i,j)}^{2}}{2\rho c_{m}}$$

where (*i*, *j*) is the position of each pixel point; ∆x and ∆y are the sampling distances (pixel size) along the *x* and *y* coordinates of the observation plane.

The reconstruction efficiency can be described as follows:

(3) $$\eta =\frac{P_{z,T}}{P_{z}}=\frac{\sum_{(i,j)\in T}p_{z,(i,j)}^{2}}{\sum_{(i,j)\in I}p_{z,(i,j)}^{2}}$$

where the target region *T* is the actual pixel set corresponding to the position of the ideal image whose amplitude is greater than 0 after 50 iterations. The entire image *I* is a collection of pixels with all the values computed.

However, in general, the assessment of acoustic field reconstruction quality transcends mere sound energy utilization and encompasses the considerations of reconstruction accuracy as well. When calculating and analyzing the efficiency, an indispensable prerequisite is to calculate the sound pressure in the region corresponding to the ideal “XDU”. Therefore, it is imperative to evaluate the reconstruction similarity of the “XDU” letter between the acoustic field of the actual target area *T* and the acoustic field of the ideal target area *D*.

Through the above modification of Equation (3), the formula of reconstructing similarity is obtained as follows:

(4) $$s=\frac{\sum_{(i,j)\in T}p_{z,(i,j)}^{2}}{\sum_{(i,j)\in D}p_{z,(i,j)}^{2}}$$

where *D* is the ideal target acoustic field area of the letter “XDU” with a non-zero amplitude.

## 3. Linear Arrays and Acoustic Holography

The two different methods of acoustic holography described above, respectively, achieved the goals of improving the accuracy of forming the target acoustic field and forming multiple complex acoustic fields at the same depth. We also designed corresponding one-dimensional linear arrays and two-dimensional planar arrays for acoustic holography under different target acoustic fields and conducted corresponding acoustic field simulations. For the linear arrays, we referenced the L7-4 linear array, with a central frequency of 5.5 MHz, 128 transducer elements, a pitch of 300 μm, and a kerf of 50 μm.

**Figure 2:**
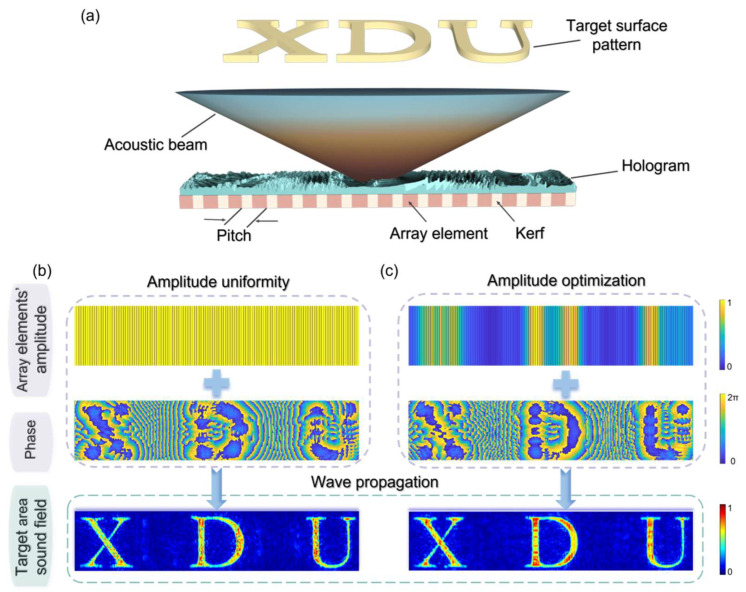
(**a**) Ideal acoustic field in the target plane, with the “XDU” portion having an amplitude of “1” and the rest being “0”. (**b**,**c**), respectively, show the target surface acoustic field distribution at a depth of 7 mm obtained by IASA after keeping the array element amplitudes the same and optimizing them.

As shown in Figure 2, the accuracy of a one-dimensional linear array acoustic field is improved by amplitude modulation. Taking the desired region “XDU” with an amplitude of 1 (others are 0) as the amplitude constraint within the target plane, as shown in Figure 2a. Both methods of maintaining uniform amplitudes for all array elements (similar to a flat probe) and optimizing array element amplitudes were employed to obtain a focused acoustic field at a depth of 7mm from the array. Both methods yielded fine pressure distributions, with the focal positions predominantly centered on the letter “XDU” and with high numerical values. However, the reconstruction efficiency for forming the acoustic field with uniform array element amplitudes was 85.97%, with a reconstruction similarity of 34.62%. After optimizing the array element amplitudes, the reconstruction efficiency was 86.51%, and the reconstruction similarity was 38.35%. Since both methods can produce focusing effects within the amplitude-constrained area, the difference in reconstruction efficiency is not significant. However, optimized focusing has better accuracy, demonstrating the clear and practical value of this method in one-dimensional linear arrays.

**Figure 3:**
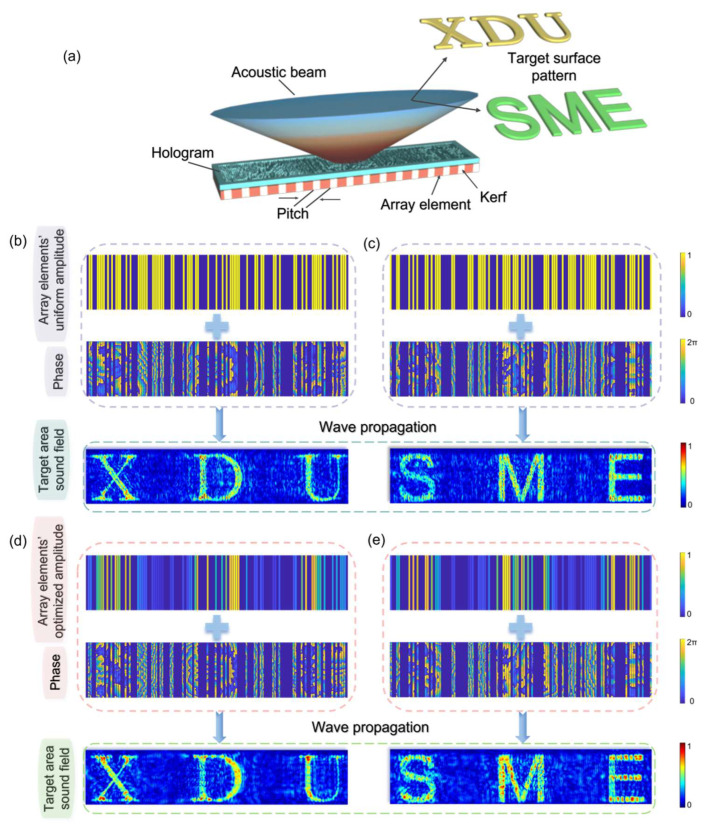
Acoustic holograms formed and target depth acoustic fields generated after random selection of array elements. (**a**) Two ideal target plane acoustic fields, with the “XDU” and “SME” portion having an amplitude of “1” and the rest being “0”. (**b**,**c**), respectively, show the acoustic hologram phase and acoustic field generated when the target acoustic fields are “XDU” and “SME” with equal excitation amplitudes for each array element. (**d**,**e**), respectively, show the final acoustic hologram phase and target acoustic field formed after amplitude optimization when the target acoustic fields are “XDU” and “SME”.

As shown in Figure 3, the one-dimensional linear array randomly selected the array elements. For selecting the target acoustic field, aside from choosing the letters “XDU”, we also selected the abbreviation of the Integrated Circuit Department of our university, “SME”, using a different font from the former to demonstrate the method’s universality. The specific patterns are shown in Figure 3a. Regarding the selection of array elements, both images correspond to a total of 32 enabled elements, but the selected elements in the two instances are completely different. This allows for the superposition of the two holograms to generate two entirely different focused acoustic fields from one complete hologram. The amplitudes of the enabled array elements are also divided into the following two categories: replicating completely identical amplitudes and advanced amplitude optimization followed by the random selection of array elements. The depth distance for both target acoustic fields is 7 mm.

From the results obtained, it is evident that both methods achieved relatively ideal target acoustic fields (Figure 3b–e), with clearly distinguishable focal patterns. In terms of the specific image reconstruction quality, the reconstruction efficiencies of the “XDU” and “SME” acoustic fields corresponding to uniform amplitudes were 60.39% and 72.40%, respectively, with the “XDU” acoustic field forming 70.25% of the entire array-enabled acoustic field. The reconstruction similarities of the formed acoustic fields were 25.07% and 21.95% for “XDU” and “SME”, respectively. The “XDU” acoustic field accounted for 72.41% of the reconstruction similarity of the entire array-enabled acoustic field. However, if array element amplitude optimization is conducted before element selection, the reconstruction efficiencies of the “XDU” and “SME” acoustic fields can be enhanced to 62.65% and 72.51%, respectively, with reconstruction similarities increasing to 28.63% and 27.76%. This indicates that even with the activation of the individual array elements to create specific acoustic fields in conjunction with acoustic holography, element optimization can still improve the precision of the formed acoustic field.

## 4. Planar Arrays and Acoustic Holography

**Figure 4:**
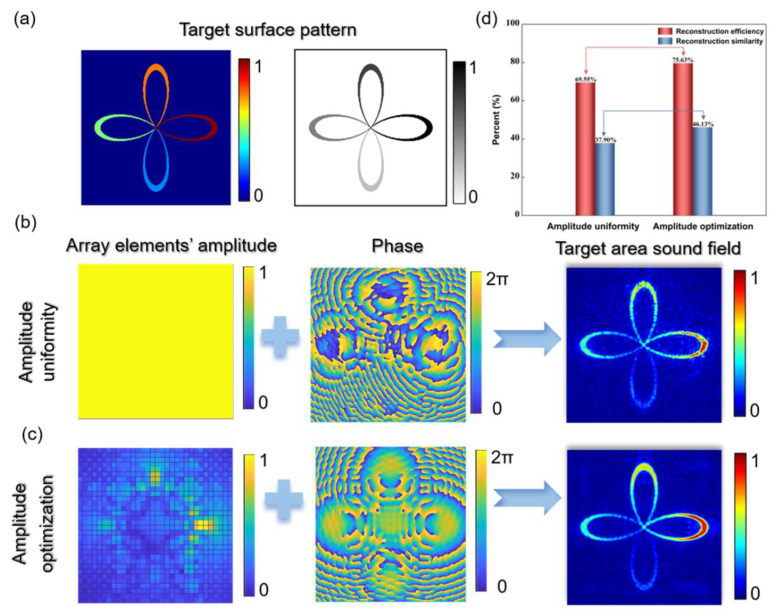
(**a**) Ideal acoustic field on the target plane, where the amplitudes represented by the four “petals” increase uniformly clockwise from the 6 o’clock direction. (**b**,**c**), respectively, show the target plane acoustic field distribution at a depth of 7mm obtained by inverse acoustic source amplitude (IASA) after maintaining uniform amplitudes for array elements and after amplitude optimization for array elements. (**d**) Comparison of reconstruction efficiency and reconstruction similarity after optimization.

We also investigated the feasibility and advantages of array element amplitude optimization and random selection based on a two-dimensional phased array [28,29]. We set the center frequency of the two-dimensional phased array to 8 MHz, with 32*32 transducer elements, a pitch of 320 μm, a kerf of 20 μm, and the target plane depth was also set to 7 mm. The array element amplitude optimization for the two-dimensional phased array is illustrated in Figure 4. Regarding the selection of the target plane amplitude pattern, we designed a “four-leaf clover” pattern, where each “leaf” represented amplitude increments in equal intervals, in a clockwise direction starting from 6 o’clock, as shown in Figure 4. When all the elements of the two-dimensional phased array were activated with equal amplitudes, it was approximately equivalent to a square flat ultrasound transducer. Utilizing the existing acoustic holography algorithms, the holographic phase shown in Figure 4b and the target plane acoustic field was obtained. The amplitude optimization of each array element after optimization is shown in Figure 4c, and the corresponding holographic phase and target plane acoustic field were obtained. The amplitude optimization for each array element after optimization is shown in Figure 4c, along with the corresponding acoustic hologram phase and target plane acoustic field. According to the different amplitude of the array elements, the color of each leaf of the four-leaf clover in the target sound field was also different, which means that we not only achieved the shape of the target image but also changed the interior of the image. Both methods achieved the desired complex focused acoustic fields. However, it is evident from the continuity of the focal clusters that the acoustic field after amplitude optimization exhibited more continuous and uniform focal lines. As shown in Figure 4d, the reconstruction efficiencies for the two results are 69.55% and 75.63%, respectively, with reconstruction similarities of 37.90% and 46.13%. Amplitude optimization significantly increased the reconstruction similarity, indicating its efficacy in enhancing the high-precision formation of complex acoustic fields.

**Figure 5:**
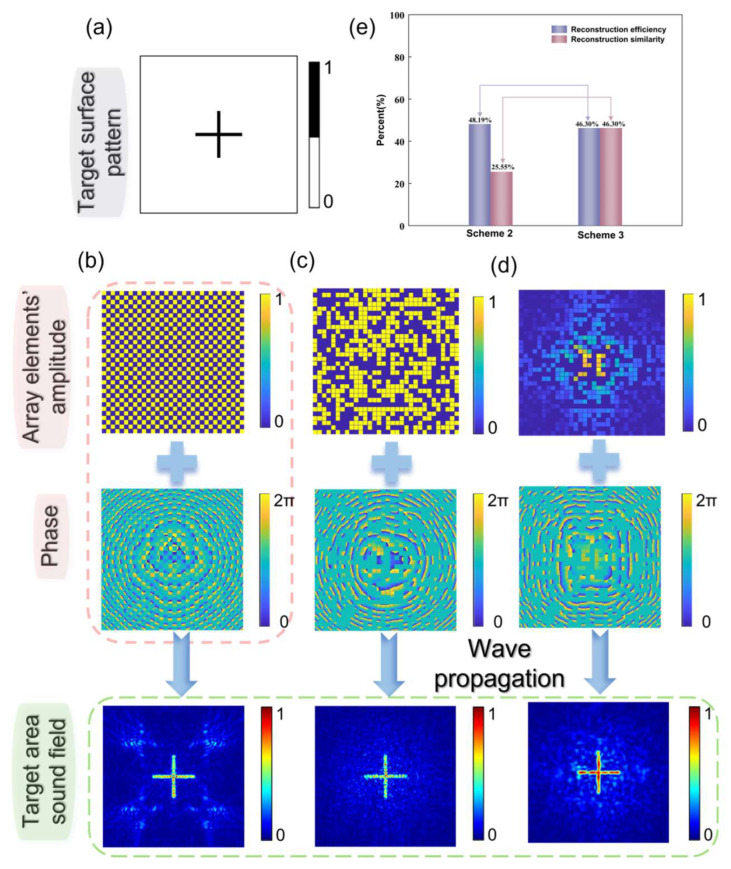
(**a**) Ideal acoustic field on the target plane. (**b**) Acoustic hologram phase and acoustic field formed when array elements are activated at regular intervals with uniform amplitudes. (**c**) Acoustic hologram phase and acoustic field formed when half of the array elements are randomly selected and activated with uniform amplitudes. (**d**) Acoustic hologram phase and acoustic field formed after array element amplitude optimization followed by random selection of 512 array elements. (**e**) Comparison of reconstruction efficiency and reconstruction similarity between two schemes.

The random selection of array elements for the two-dimensional phased array is depicted in Figure 5. For the target acoustic field, we designed two vertically intersecting rectangular bars, each measuring 3mm in length and 2.2mm in width, as shown in Figure 5a. Four selection methods for array element amplitudes were compared. These methods are as follows: Scheme 1: Half of the array elements are activated with uniform amplitudes at regular intervals, totaling 512 elements (Figure 5b); Scheme 2: 512 array elements are randomly selected and activated with uniform amplitudes (Figure 5c); and Scheme 3: Array element amplitude optimization is performed first, followed by the random selection of 512 array elements (Figure 5d).

It is evident that Scheme 1 will result in noticeable artifacts on the target plane, which is undesirable and underscores the importance of random array element selection. For Scheme 2 and Scheme 3, their reconstruction efficiencies are 48.19% and 46.30%, respectively, with reconstruction similarities of 25.55% and 46.30% (Figure 5e). This indicates that amplitude optimization in a two-dimensional phased array may slightly reduce the effectiveness of image reconstruction but can help improve the accuracy of reconstructing the target acoustic field. Additionally, observations show that the focal positions of the acoustic field formed after amplitude optimization are more continuous, enhancing the practical applicability of some complex acoustic fields.

**Figure 6:**
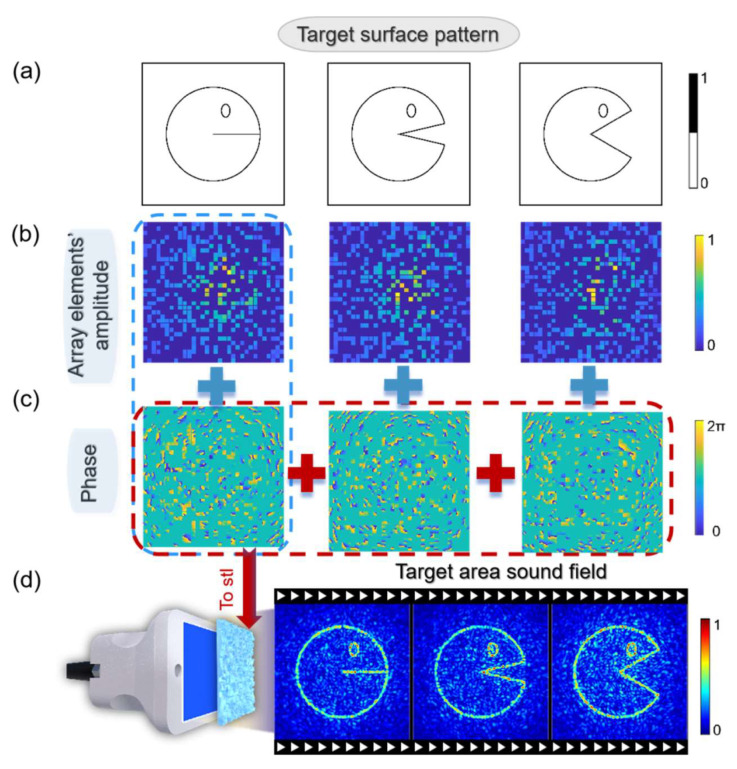
(**a**) Ideal acoustic field for each target plane. (**b**) Distribution of element amplitudes optimized for each specific array element. (**c**) The corresponding phase of acoustic holography after optimization. (**d**) Dynamic acoustic field animation achieved by creating an acoustic hologram by superimposing the three phases and using a phased array.

As the random selection of array elements allows for some elements to focus the entire acoustic field, multiple different acoustic fields can be stored in the same acoustic hologram phase. With a sufficient number of array elements, it is possible to achieve a frame-by-frame animation effect similar to film projection. As shown in Figure 6a, we drew three different styles of Pac-Man characters. We divided all array elements into three groups (with one element not activated) and performed array element amplitude optimization. This enabled us to achieve changes in the Pac-Man images by altering the array elements (Figure 6b–d).

## 5. Discussion and Conclusions

In this study, we provide a new method for acoustic field regulation, integrating the flexible control of ultrasonic arrays with the precise control of acoustic fields via acoustic holography. This method not only achieves a more precise control of target acoustic fields but also allows for the creation of multiple different complex acoustic fields from a single acoustic hologram by randomly selecting array elements. We performed acoustic holography design and simulation validation for a linear array, with a center frequency of 5.5 MHz and 128 elements, and a two-dimensional planar array, with a center frequency of 8 MHz and 32*32 transducer elements. We objectively assessed the quality of the formed acoustic fields using the proposed metrics of reconstruction efficiency and reconstruction similarity. For the linear array, optimizing the array element amplitudes resulted in improvements in both reconstruction efficiency and reconstruction similarity. By selectively activating array elements, we were able to control multiple different styles of acoustic fields (XDU and SME) at the same target depth from a single acoustic hologram. With the addition of array element amplitude optimization, reconstruction efficiency increased to 62.65% and 72.51% while reconstruction similarity improved to 28.63% and 27.76%. For the planar array, optimizing the array element amplitudes also enhanced reconstruction efficiency and reconstruction similarity. Furthermore, randomly activating array elements achieved ideal acoustic fields with fewer elements while addressing potential artifact issues. It was also observed that using array element amplitude optimization might slightly reduce reconstruction efficiency, but it can significantly enhance reconstruction similarity. By continuously and randomly activating non-repetitive array elements, we can achieve ongoing changes in the acoustic field, thereby creating the “Pac-Man” animation effect.

Compared with general harmonic ultrasound imaging, acoustic holography images have clear advantages and disadvantages. Acoustic holography provides enhanced spatial resolution, enabling the capture of extremely fine details of the imaged object and better detection of small tumors or lesions. It offers improved contrast, facilitating easier discrimination between normal and abnormal tissues. The technique also has remarkable 3D imaging capabilities, valuable for surgical planning and providing a comprehensive understanding of anatomy. Additionally, it can reduce artifacts through sophisticated signal processing techniques. However, acoustic holography is a complex technique with significant computational demands, limiting real-time imaging capabilities and increasing processing time. It is sensitive to environmental factors, requiring careful calibration. Like general harmonic ultrasound imaging, it may have limited penetration depth.

The aforementioned research will successfully provide theoretical support for subsequent specific experiments, broadly expanding the application methods of acoustic holography. It is anticipated to be applied in more complex and even three-dimensional precise and dynamic acoustic field control in the future, offering more insights for applications such as acoustic tweezers and neural modulation. This approach could advance traditional biologic therapies, enabling more precise drug delivery or cell control [30,31]. In addition, the control of a focused sound field with high resolution can also promote the development of the traditional bioimaging field [32].

## Data Availability

The raw data supporting the conclusions of this article will be made available by the authors on request.
